# Worm-Based Diagnosis Combining Microfluidics toward Early Cancer Screening

**DOI:** 10.3390/mi15040484

**Published:** 2024-03-31

**Authors:** Yutao Shi, Chen Cui, Shengzhi Chen, Siyu Chen, Yiheng Wang, Qingyang Xu, Lan Yang, Jiayi Ye, Zhi Hong, Huan Hu

**Affiliations:** 1 Zhejiang University-University of Edinburgh Institute (ZJU-UoE Institute), Zhejiang University School of Medicine, International Campus, Zhejiang University, Haining 314400, Chinasiyu.21@intl.zju.edu.cn (S.C.); qingyang.19@intl.zju.edu.cn (Q.X.);; 2 Zhejiang University-University of Illinois Urbana-Champaign Institute (ZJU-UIUC Institute), International Campus, Zhejiang University, Haining 314400, China

**Keywords:** *C. elegans*, early cancer diagnosis, microfluidics, chemotaxis assays

## Abstract

Early cancer diagnosis increases therapy efficiency and saves huge medical costs. Traditional blood-based cancer markers and endoscopy procedures demonstrate limited capability in the diagnosis. Reliable, non-invasive, and cost-effective methods are in high demand across the world. Worm-based diagnosis, utilizing the chemosensory neuronal system of *C. elegans*, emerges as a non-invasive approach for early cancer diagnosis with high sensitivity. It facilitates effectiveness in large-scale cancer screening for the foreseeable future. Here, we review the progress of a unique route of early cancer diagnosis based on the chemosensory neuronal system of *C. elegans*. We first introduce the basic procedures of the chemotaxis assay of *C. elegans*: synchronization, behavior assay, immobilization, and counting. Then, we review the progress of each procedure and the various cancer types for which this method has achieved early diagnosis. For each procedure, we list examples of microfluidics technologies that have improved the automation, throughput, and efficiency of each step or module. Finally, we envision that microfluidics technologies combined with the chemotaxis assay of *C. elegans* can lead to an automated, cost-effective, non-invasive early cancer screening technology, with the development of more mature microfluidic modules as well as systematic integration of functional modules.

## 1. Introduction

Cancer poses a significant global health challenge and remains one of the leading causes of death. According to recent statistics published in the *CA: A Cancer Journal for Clinicians*, approximately 2 million new cancer cases and over 0.6 million cancer-related deaths were predicated in the year 2023 [1]. Timely cancer detection, before the tumor migrates to distant tissues (stage IV), is crucial in reducing the risk of cancer-related mortality. For instance, the 5-year survival rate for colorectal cancer diagnosed at stage III is 73%, whereas it significantly drops to 16% when diagnosed at stage IV [2]. This highlights the critical role of early detection in improving patient outcomes. However, only limited types of cancer, such as cervical, colorectal, and breast cancers [2], among many cancer types, can use population-wide screening. Therefore, further research is needed to enhance our understanding of clinical diagnosis and treatment strategies to enable early detection for more types of cancers.

Currently, early cancer screening primarily relies on blood-based cancer markers and endoscopy procedures. Carbohydrate antigen (CA 19–9) and carcino-embryonic antigen (CEA) are commonly used biomarkers in clinical practice. However, their diagnostic accuracy is relatively low due to susceptibility to interference from benign conditions, such as pancreatitis and jaundice [3]. Recent studies have highlighted the potential of various molecules in serum, including mRNA, microRNA, enzymes, and metabolites, as cancer biomarkers [4]. Nonetheless, further validation is required to establish their clinical significance [5]. In addition to biomarkers, endoscopy plays a vital role in the detection of early gastrointestinal cancers by allowing visualization and collection of biopsy samples for pathological examination. However, endoscopy procedures are complex, invasive, and costly [6], limiting their accessibility to a small fraction of the population each year. Consequently, there is a pressing need to develop reliable, non-invasive, and cost-effective methods for early cancer screening, as such advancements hold the potential to revolutionize cancer diagnosis and treatment.

Urine tests have gained recognition as promising non-invasive tools for cancer diagnosis [7]. For example, Leyten et al. demonstrated that a combination of urine PCA3 and TMPRSS2 as prostate-specific markers exhibited 86.7% sensitivity and 51.4% specificity [8]. Remarkably, several studies have shown that species such as *Caenorhabditis elegans* (*C. elegans*), canines, and mice can detect odors from urine samples of cancer patients with an accuracy exceeding 80% [9,10,11]. However, the attention span of dogs diminishes in hot testing environments [12], and the utilization of large animals, such as dogs, for detection tools poses challenges in terms of time and economic costs for commercialization. Considering the relatively easier and cost-effective maintenance of *C. elegans*, as well as its sensitive chemosensory system, *C. elegans* is emerging as an attractive platform for early cancer screening.

*C. elegans*, a simple model organism widely used in various research fields, including olfactory investigations, possesses over 1500 G-protein-coupled receptors (GPCRs) located in the cell membrane. These receptors are essential in translating extracellular signals into physiological effects [13], making *C. elegans* highly susceptible to the environment.

Hirotsu et al. reported the pioneering work of utilizing the chemosensory abilities of *C. elegans* for cancer diagnosis [14], which initiated a novel strategy for detecting early cancer and studying cancer biomarkers. They discovered that wild-type *C. elegans* exhibited attractive chemotaxis toward cancer cell secretions, tissues, and urine samples from patients, with a specificity of 95.8% based on 242 samples. Hence, *C. elegans* holds promise as a significant tool for large-scale cancer screening in the future. Worm-based diagnosis currently detects 15 types of cancers, including breast, prostate, stomach, and esophageal cancers, with over 500,000 individuals having undergone this testing to date (https://lp.n-nose.com, accessed on 21 March 2024). This method exhibits high sensitivity, effectively screening for early-stage cancers, thereby aiding in the timely identification of cancer risk for patients. Additionally, literature reports suggest that *C. elegans* demonstrate distinct behavioral changes before and after surgical removal of cancer [15], implying potential application value in postoperative evaluation. The chemosensation process in *C. elegans* involves synchronization, chemotaxis, immobilization, and counting. However, traditional methods suffer from low throughput, and the tested substances are easily contaminated by other odors, leading to false-negative results [16]. Microfluidics, a technology that enables precise control of fluid flow at the micron scale, has emerged as a powerful tool for optimizing the chemosensation process, enhancing throughput and reliability [17]. Notably, microfluidics technology has enabled behavioral analysis under chemical stimulation and rapid synchronization. Each step of the chemotaxis experiment can match a microfluidic function module. However, a systematic review summarizing their relationship and further microfluidic system integration is missing.

This review begins by introducing the current standard procedures of *C. elegans*-based chemosensation methods for early diagnosis using traditional technology. Subsequently, we summarize the state-of-the-art progress in using microfluidics to enhance key processes in *C. elegans*-based chemosensation. The advantages and disadvantages of microfluidics-based lab-on-a-chip technologies compared to traditional methods are discussed. Finally, we envision several promising technical routes for applying microfluidics to improve early cancer detection based on *C. elegans*.

## 2. Standard Worm-Based Diagnosis Procedure Using Chemotaxis Behavior Assays

**Figure 1:**
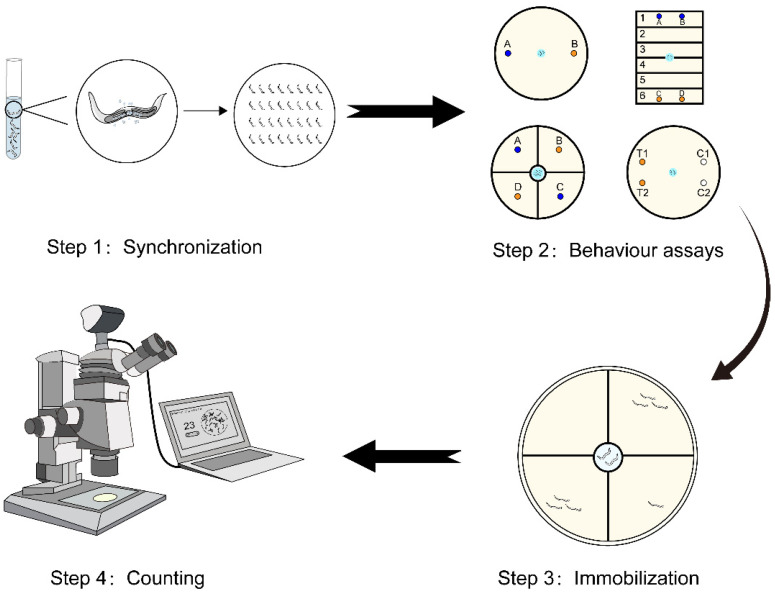
The protocol of a standard worm-based diagnosis procedure. *C. elegans* at the L4 developmental stage is firstly selected through the synchronization step. Then, selected *C. elegans* are placed at the center of the plate and a certain period of time is allowed for *C. elegans* to move to the regions. Then, optical microscopy is employed for counting *C. elegans* in regions containing different chemicals to evaluate the chemotaxis behavior.

Figure 1 shows the chemotaxis behavior assays of *C. elegans* toward urine samples employed traditionally for early cancer detection [14]. In this traditional method, urine samples and ultra-pure water are spotted on opposite ends of the assay plate, with an anesthetic applied at these spots as well. *C. elegans* at the L4 developmental stage are then placed at the center of the plate. *C. elegans* can move freely starting from the center point. The volatile odor of the sample either attracts or repels the *C. elegans* [18]. When worms arrive at the urine sample area or ultra-pure water area, they are immobilized by an anesthetic applied at these spots, facilitating the counting of individuals within each area. As shown in the first image of “Step 2” in Figure 1, the chemotaxis index (CI) is defined as:

$$\mathrm{CI}=\frac{A-B}{A+B}$$

where A and B denote the counts of *C. elegans* in the test sample and control sample areas, respectively. CI is subsequently calculated to determine the level of attractiveness of *C. elegans* toward patient samples, thereby indicating the risk of cancer [19]. Until now, the effectiveness of the worm-based diagnosis has been demonstrated across various types of cancer, as summarized in Table 1. The purposes, advantages and disadvantages of standard worm-based diagnosis procedure are summarized in Table S1.

### 2.1. Developmental Stage Synchronization of C. elegans

The initial step of synchronizing the developmental stage of *C. elegans* is crucial for longitudinal studies and minimizing variability [24]. The lifecycle of *C. elegans* has three stages: embryonic development, larval stages (L1–L4), and the adult stage [18]. The traditional chemotaxis assay predominantly employs *C. elegans* at the L4 larval stage due to their relatively mature chemosensory system [25]. There are two main methods for stage synchronization: manual picking and chemical sterilization (“bleaching”). Scientists either manually select and transfer gravid worms onto new agar plates, allowing them to produce synchronized eggs, or employ bleaching using solvents, such as alkaline hypochlorite solution (NaOH 0.5 M, NaClO~0.8%), to sacrifice gravid worms and obtain eggs [26,27]. These eggs are subsequently cultured at 20 °C under well-fed conditions for 37 h to obtain populations of L4 *C. elegans*.

### 2.2. Methods Used for Behavior Assays

Preclinical studies have demonstrated that the average CI value detected from cancer patients (e.g., cancer tissue, urine, and blood serum) is higher than that of normal individuals, indicating an increased attraction of *C. elegans* toward cancer-related metabolites [14]. Therefore, the CI value can serve as a potential clinical indicator for early cancer detection.

**Figure 2:**
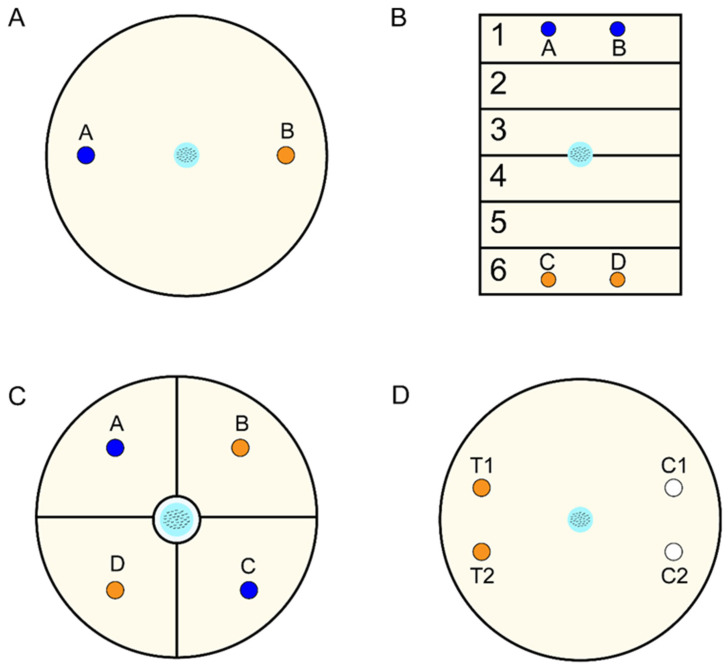
General plate design for behavior assays. (**A**) A fundamental two-point chemotaxis plate for distinguishing attraction or repellence (A: Control group; B: Experimental group). (**B**) Avoidance behavior assays conducted on a square plate (A.B: Control group; C,D: Experimental group; 1–6 zones: Scoring regions). (**C**) An enhanced four-point chemotaxis plate for discriminating between attraction and repellence (A,C: Control group; B,D: Experimental group). (**D**) Schematic of the pond assay for sensory systems for behavior assays (T1,T2: Experimental group; C1,C2: Control group).

To ensure the accuracy and reliability of the CI value, the design of the assay plate requires a careful design. In 1993, Bargmann utilized 10 cm diameter, round tissue culture dishes to evaluate the attraction or repellence of *C. elegans* toward volatile chemicals [19], as shown in Figure 2A. The dish was divided into two areas, with test samples placed on one side and corresponding control samples on the other side. Approximately 100–200 L4 to adult *C. elegans* were then positioned at the center of each dish, and the assay results were obtained within 1 h. This method offered advantages such as minimal sample requirements (1–2 μL), straightforward processing, and a relatively short testing time. However, issues such as ambiguous demarcation lines and a significant number of immobile *C. elegans* during the test period contributed to counting inaccuracies.

Subsequent studies addressed these issues. Troemel et al. devised an avoidance assay on a square plate, divided into six distinct sectors, to assess the long-range avoidance of volatile repellents [28], as shown in Figure 2B. This design, compared to round plates, minimized confusion caused by odors thanks to increased distances between the repellent and control regions. Moreover, Margie et al. developed a modified chemotaxis assay using two opposite quadrants, labeled as “test” and “control” [18], as shown in Figure 2C. This approach optimized the starting position of the worms in relation to the control and test areas by introducing four quadrants and creating alternating patterns of test and control regions. Consequently, this method reduced the likelihood of worms following convoluted paths by passing through other individuals. Moreover, marking a circle around the starting location allowed for the exclusion of immobile worms and prevented any bias. However, one drawback of this method is the potential confusion of *C. elegans* due to the mixed scents of the “test” and “control” regions, which are in close proximity to each other. More recently, Suzuki et al. proposed a pond assay for sensory systems in behavior assays [29], as shown in Figure 2D. This approach involved creating small holes by removing agar from some circular regions of the plates, into which the test or control solution was injected. Once *C. elegans* fell into these holes, they could be trapped without the need for anesthetic. This method enabled the detection of samples even at extremely low concentrations, such as 6- or 7-step dilutions of diacetyl.

### 2.3. Classic Immobilization Methods

After *C. elegans* moves to the target area, it is necessary to immobilize the worms for subsequent imaging and counting. Previous studies have employed anesthetic reagents to inhibit physiological functions or utilized physical techniques to restrict movement [30].

Sodium azide, a potent reversible inhibitor of mitochondrial respiration, is widely used as an anesthetic [31]. Another chemical, 1-phenoxy-2-propanol (1P2P), has been indicated to reduce neural activity by inhibiting action potentials with reversibility [32]. Although these chemicals are effective for immobilization, potential sustained stress responses have been reported [30,33], which can be detrimental to subsequent generations of *C. elegans*. In addition to chemicals, researchers have proposed a strategy that involves a short incubation at 4 °C, known as cold shock [34]. However, exposure to cold temperatures has been found to cause tissue damage. Although the damage may not be immediately fatal, if significant enough to be irreparable, it can still result in delayed lethality [34].

Methods based on physical techniques involve introducing physical constraints through the use of glue or nanoparticles. Kim et al. demonstrated a technically simple immobilization method utilizing polystyrene nanoparticles and agarose pads [35]. They placed one or more washed *C. elegans* and a suspension of polystyrene beads on a cooled agarose pad. This method limits movement by increasing the friction between *C. elegans* and the solid surfaces. However, complete immobilization of *C. elegans* was not achieved, as the head and tail retained limited freedom [30]. Kyra et al. used a polyethylene glycol (PEG) hydrogel to immobilize *C. elegans* [36]. They trapped the worms using a hydrogel precursor solution, which was then crosslinked by exposure to ultraviolet (UV) light for a few seconds. This strategy is efficient in immobilization and can be performed at a range of temperatures. However, exposure to UV light may cause minimal spectral interference and DNA damage [36].

### 2.4. Automatic Worm Detection Systems

To quantify the value of CI and confirm the behavioral responses elicited by olfactory cues, the final step is to count *C. elegans*. In recent years, significant progress has been made in both computer vision and machine learning, enabling the development of automated detection systems with substantial improvements in speed and accuracy [37,38]. In 2022, Crombie et al. introduced an automated approach called chemotaxis-cli, implemented in Python, for scoring CI [39]. This script can automatically identify the boundaries of assay plates and filter out non-nematode objects. By measuring the area of worms within each quadrant, this method eliminates errors caused by clustered worms. Similarly, Shinichiro Mori et al. developed an automated worm detection system based on a deep neural network (DNN) with multi-class classification (MCC) for worms [40]. The MCC approach achieved an average accuracy of 0.90 in detecting over 100 worms, with false identifications mainly attributed to the non-maximum suppression (NMS) algorithm. These automated counting tools reduce subjective judgments from human operators and enhance effectiveness, although the challenge of tallying high-throughput data remains.

In a short summary, continuous advancements in chemotaxis behavior assays toward achieving high accuracy, non-toxicity, and automation have been generated. However, there is still a lack of systematic tools that can support not only fundamental research but also clinical applications, while maintaining high throughput and low cost.

## 3. Microfluidic Systems for High-Throughput Cancer Screening Using *C. elegans*

**Scheme 1:**
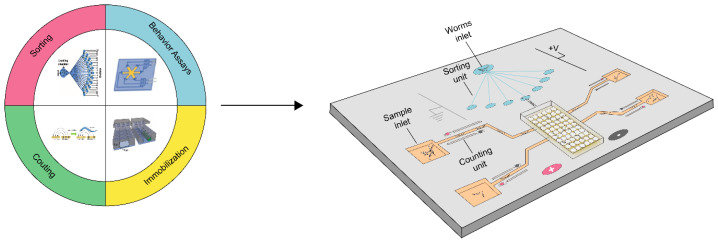
Overview of microfluidic systems for high-throughput cancer screening.

Microfluidic technology, also known as lab-on-a-chip technology, has emerged as a promising platform since its inception in the early 1990s [41,42,43]. It features small channels, minimal reagent and sample consumption, automation, laminal flow, etc. [44]. These features enable lab-on-a-chip technology to be increasingly applied in various fields of biology. In 2004, Bargmann pioneered the use of microfluidics in worm research and subsequently developed a series of worm-based microfluidic systems [45]. For example, Chung et al. employed microfluidics for neurobiology studies [46], while Yang et al. utilized it for toxicology research, specifically for evaluating in vivo antimicrobial activity of natural compounds using a whole-animal infection model [47]. Carr et al. also harnessed microfluidics for drug screening purposes [48]. Overall, microfluidic technology has proven to be a powerful tool in advancing biomedical research, enabling precise and efficient experimentation and analysis. In this section, we will summarize the progress of microfluidics technology that has improved or has the potential to improve the efficiency of each key process in cancer diagnosis using *C. elegans*, as shown in Scheme 1. The purposes, advantages and disadvantages of microfluidic systems for high throughput cancer screening are summarized in Table S2.

### 3.1. Microfluidics for Sorting C. elegans

In traditional biological laboratories, manual picking and chemical sterilization are commonly employed due to ease of use [24]. However, these methods suffer from low throughput, substantial workforce, and heavy reagent consumption. Furthermore, the use of bleach can adversely affect the physiology of *C. elegans* [49]. Fortunately, with the integration of mechanical and electrical microfluidic components, worm sorting technology has matured, effectively addressing the challenges posed by traditional synchronization methods [50,51,52,53,54,55,56,57,58,59,60,61]. The microfluidic channel is designed to accommodate the size of the worm, enabling precise sorting [44]. Additionally, the microfluidic system exhibits high compatibility and can seamlessly integrate with other microfluidic components to perform additional operations [62].

**Figure 3:**
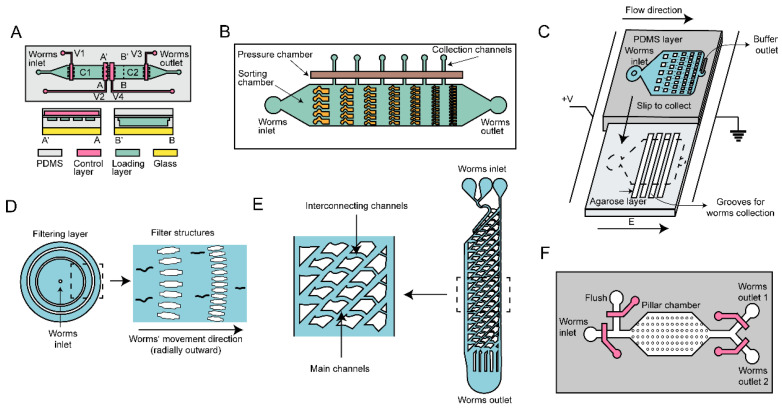
Size-based microfluidic scheme for sorting. (**A**) Diagram depicting a microfluidic device utilizing externally controlled PDMS channels for specific worm sorting based on channel sizes controlled by pressure(C1, C2: Worm chambers; V1-4: Pressure valves; A-A’, B-B’: Lines of cross-sectional views). (**B**) Schematic representation of the microfluidic sorting device based on diode sizes. (**C**) A microfluidic chip with size gradient microfilters adapted to different stages of nematodes. (**D**) Elaboration on the adjustable PDMS filter structures, including stage size-related filter array structures, which are designed for size-based sorting. (**E**) “Smart” mazes consisting of a network of intercommunicating channels, which are designed to guide worms of various sizes along distinct vertical trajectories. (**F**) A microfluidic device incorporating a pillar arrays switch for sorting of worms by leveraging fluid pressure.

The first type of sorting approach is size-based physical flow filtration, widely employed in microfluidics. This method can sort *C. elegans* just based on size without altering their physiological effects, featuring simplicity and convenience. Channels with varying channel diameters serve as efficient filters for this purpose. Dong et al. employed PDMS to construct filter structures with adjustable sizes [50], as shown in Figure 3A. By controlling the pneumatic pressure in the control layer, the membrane between the control layer and load layer deforms, and thus regulates the cross-sectional area, allowing specific developmental stages of worms to pass through. This method successfully isolates an average of 3.5 worms per second from mixed populations consisting of L1 to L4 larvae and adults. Furthermore, the stacking of fixed channels with different diameters enables stage differentiation. Yang et al. designed a series of microfluidic diode structures and interconnected them to achieve stage-specific sorting [51], as shown in Figure 3B. The basic unit of the channel consists of a curved pipe section and a straight channel section that controls the passage of nematodes in one single direction. By maintaining a slow buffer flow rate, the sorting efficiency is preserved, reducing the impact of debris clogging. Similarly, Wang et al. employed channel cascades for sorting purposes [52], as shown in Figure 3C. They improved the sorting accuracy and efficiency by optimizing buffer flow rates and electric fields. To simplify size filtering operations for broader applications, Atakan et al. developed a hand-held tool based on channels of different diameters, which is reusable and does not require electrical controls, offering greater convenience in sorting [53], as shown in Figure 3D. Additionally, many sorting methods combine size-based and behavior-based differentiation. In 2011, Solvas et al. developed a “smart maze” consisting of main and interconnected channels capable of sorting four to five worms per second [54], as shown in Figure 3E. The main channel allows for the enrichment of adults, while larvae are directed into smaller channels through specially designed connections based on diameter and orientation. However, this method only distinguishes between adults and larvae and cannot be used to sort worms at different larval stages. Ai et al. addressed this limitation by designing a micropillar array element [55], as shown in Figure 3F. The element allows smaller worms to pass through the columns via sudden pressurization, while trapping larger worms. Four column arrays with different spacing, designed based on the sizes of *C. elegans* at different stages, are applied to the same element, enabling accurate separation of worms at different developmental stages.

**Figure 4:**
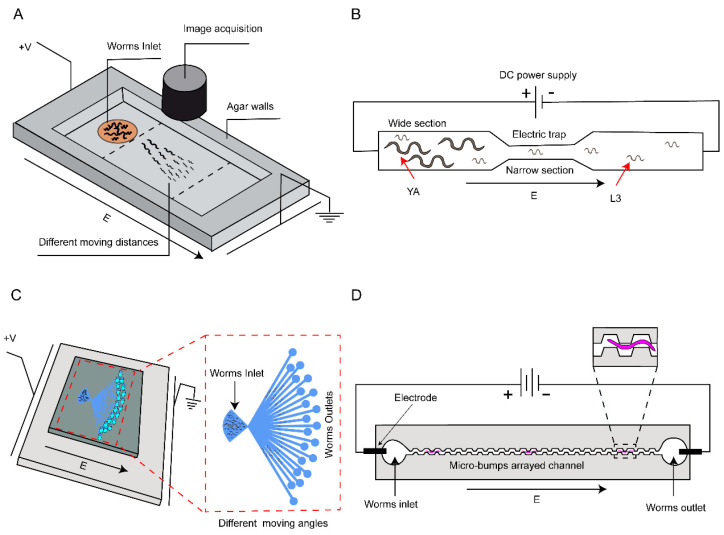
Electric field-based microfluidic scheme for sorting. (**A**) A microfluidic device using an electrophoresis chamber for sorting based on the electrotaxis of *C. elegans*. (**B**) Diagram illustrating a single electric trap designed to allow different stage worms to pass through the narrow section according to the matched electric field. (**C**) A microfluidic system with an electric field to manipulate worms of varying sizes, effectively diverting them at distinct angles. (**D**) An electrotaxis experimental setup, integrating micro-bump arrayed channels, is employed to enhance locomotion speeds and discriminate between worms at different developmental stages.

The second type of sorting approach employs the behavior differences of nematodes at different stages in response to an applied electric field. Electrotaxis, a phenomenon where biological targets swim toward a specific polarization in an electric field [63,64], has also been utilized for sorting *C. elegans*. In 2011, Manière et al. employed classical electrophoresis to spatially sort *C. elegans* based on their movement speed at different stages under an electric field [56], as shown in Figure 4A. Building upon this, Rezai et al. exposed worms to localized high electric fields and classified them based on their distinct electrical contact responses [57], as shown in Figure 4B. While this method achieves a throughput of up to 78 worms per minute, the sorting accuracy is not optimal. To enhance the sorting accuracy, Wang et al. designed a microfluidic device including sorting channels with different angles [58], as shown in Figure 4C. The design rationale is based on the observation that the angle of movement of *C. elegans* is proportional to the strength of the applied electric field. In addition to sorting worms at different developmental stages, the device can also separate males and mutants, although the sorting purity is not sufficiently high. Han et al. designed a sorting channel featuring continuous protruding hexagonal columns [59], as shown in Figure 4D. This design enables the continuous sinusoidal motion of *C. elegans*, and by adjusting the parameters of the protrusions, the velocity of nematodes at a specific developmental stage can be optimized for sorting purposes. An electric field is applied in the channel to leverage the electrotaxis of worms and promote their directional movement.

**Figure 5:**
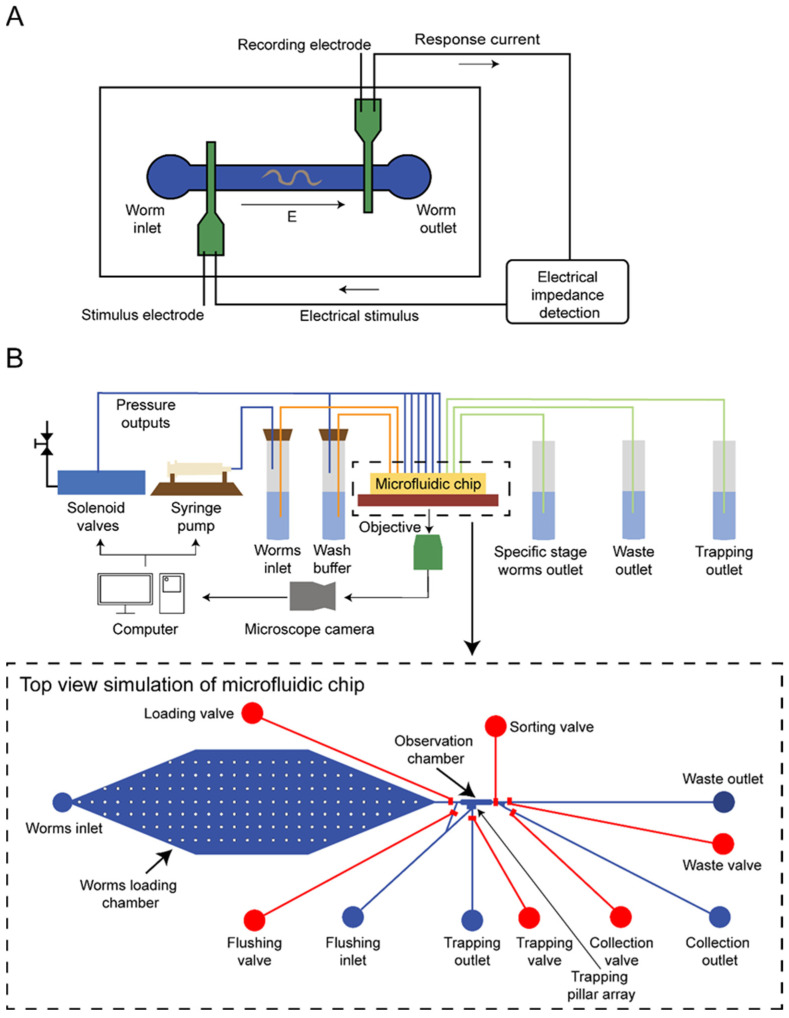
Sensor-based microfluidic scheme for sorting. (**A**) A microfluidic device enabling precise identification and efficient sorting of distinct life stages in *C. elegans* through the utilization of microfluidic impedance cytometry. (**B**) A dual-layer microfluidic device for worm sorting based on advanced visual detection and sizing algorithms.

The third type of approach combines a sensing module with a microfluidic valve to separate the nematodes. Unlike the previous two methods that integrate differentiation and separation across multiple components, this approach first employs sensing to distinguish nematodes at different stages and subsequently selects nematodes using separators. Chen et al. discovered a linear relationship between the length of *C. elegans* and the cubic root of the electrical impedance spectroscopy (EIS) signal amplitude [60], as shown in Figure 5A. The nematode passes through the impedance detection region of the microfluidic channel, and the resulting signal is amplified and received by the impedance microscope. A dedicated computer program processes these signals to control the opening and closing of a dichotomous valve at the end of the pipeline. Dong et al. employed an automated size-based classification method [61], as shown in Figure 5B. After a single nematode is pushed from the loading chamber into the observation chamber by the liquid, an image processing algorithm extracts the boundary, dorsal, and ventral sides of the nematode. Subsequently, the length and width of the nematode are calculated, and the target nematodes are washed out of the observation chamber. While this method may not achieve the highest speed and success rate, it offers the advantage of accurately measuring the size of nematodes.

### 3.2. Microfluidic Systems for Behavior Assays of C. elegans

Microfluidic technology has emerged as a promising alternative to the conventional agar plate-based approach for behavior assays of *C. elegans*. Agar plates, although easy to prepare and widely available [18], have limitations when applied for early cancer screening. These limitations include low throughput, susceptibility to environmental variations, and the potential for human operator error. Additionally, the slow diffusion of chemicals on agar plates leads to a time-consuming process for establishing specific concentration gradients [65]. Another drawback of traditional agar plates is the lack of clear demarcation, which can result in interactions between *C. elegans* and compromise the results’ accuracy [66]

In contrast, microfluidic systems offer precise control over fluid flow using micro-pumps and micro-valves, enabling the generation of time- and space-controlled concentration gradients in microchannels [67,68,69]. Microfluidic channels are designed with appropriate diameters to allow a single worm to pass through without encountering specific interactions [70,71]. Moreover, the miniaturization of integrated components in microfluidic systems enables increased throughput, while reducing labor and consumables’ usage [68].

**Figure 6:**
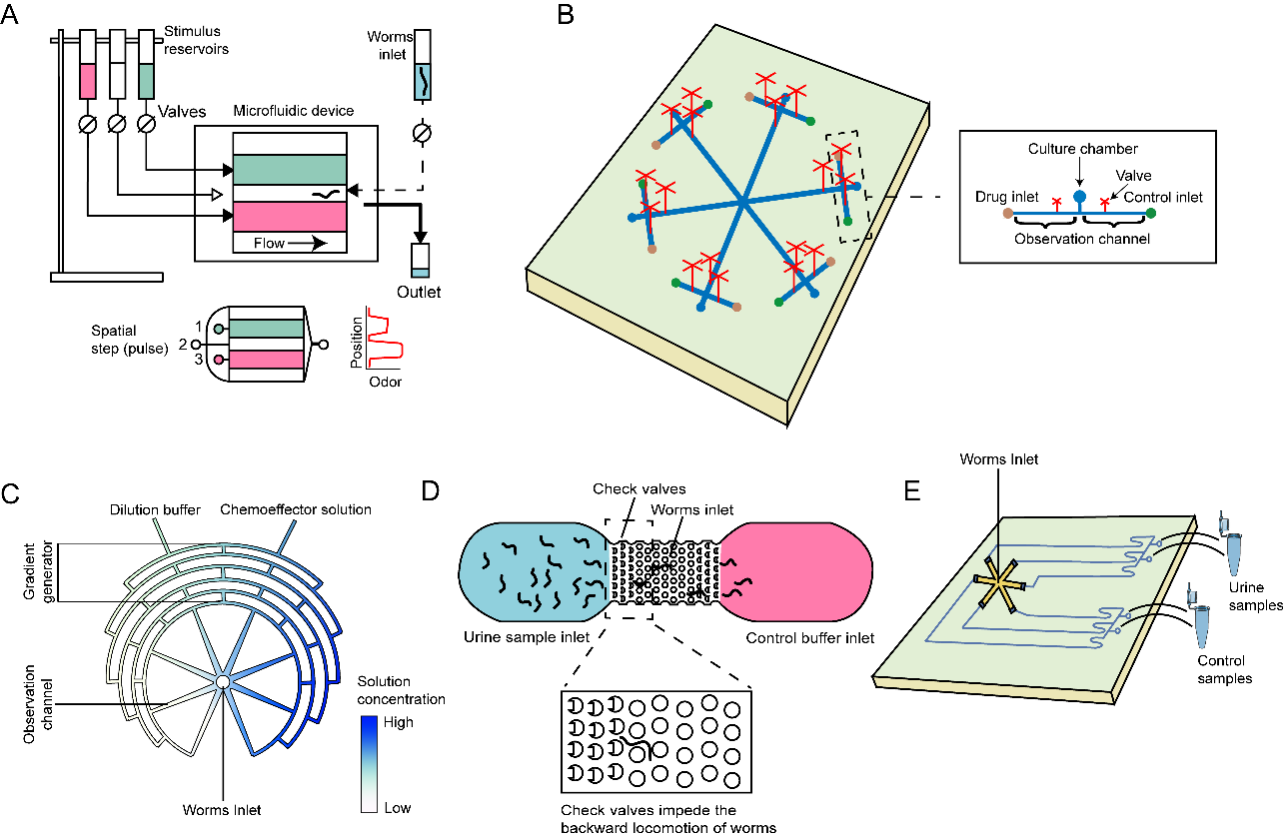
Schematic illustrations of microfluidic systems employed in behavior assays of *C. elegans*. (**A**) A microfluidic device for behavior assays under precise spatiotemporal stimulation patterns. (**B**) Schematic diagram of an integrated microfluidic chip for chemotaxis assays and chemotaxis-defective mutant screening. (**C**) An eight-channel microfluidic device for chemotaxis analysis in *C. elegans*, which can rapidly create progressive chemical concentration gradients. (**D**) A microfluidic device schematic for cancer detection in *C. elegans*, avoiding the reverse locomotion by check valves. (**E**) A hexagonal worm-based biosensor applied for the detection of breast cancer.

In 2011, Albrecht and Bargmann et al. introduced a novel microfluidic platform consisting of micro-post arrays and dendritic microfluidic networks [67], as shown in Figure 6A. This platform enables the application of three distinct stimulation modes (temporal pulses, spatial stripes, and a linear concentration gradient) to *C. elegans*. However, this method is somewhat impractical for general biological assays, as it relies on advanced external equipment for characterizing chemosensory behavior. Yang et al. developed an integrated microfluidic system capable of conducting chemotaxis assays or rapidly screening chemotaxis-defective mutants [68], as shown in Figure 6B. With the support of a micro-metal-pin valve and micro-pump, this automated device eliminates the need for anesthesia to immobilize the worms and can detect up to six compound samples simultaneously in just 40 min. In addition, Hu et al. proposed a chemotaxis system comprising eight microchannels, each featuring a gradual chemical concentration gradient [69], as shown in Figure 6C. This design maintains a consistent flow rate of liquid in each channel, thereby minimizing the impact of liquid rheological effects on *C. elegans*.

In addition to the microfluidic systems designed for compound testing, there have been studies exploring the direct detection of cancer cells or urine samples using *C. elegans*. Shiga and colleagues optimized the pillar periodicity and successfully developed a microfluidic channel integrated with check valves [72], as shown in Figure 6D. This innovative approach improved the locomotion speed of *C. elegans* and effectively prevented reverse locomotion, leading to enhanced accuracy in early cancer diagnosis. Similarly, Zhang and co-workers developed a worm-based microfluidic biosensor that utilized an agar-covered hexagonal channel [16], as shown in Figure 6E. When exposed to samples of breast cancer cell populations, *C. elegans* displayed distinct chemotaxis preferences, enabling real-time assessment of breast cancer status. This approach serves as a compelling example of applying microfluidic systems for high-throughput detection of metastatic cancer phenotypes.

### 3.3. Microfluidic Approaches for Immobilization

Microfluidic approaches also could overcome many limitations of traditional methods for immobilizing *C. elegans* [30,33,73,74]. Although conventional techniques, such as the use of nanoparticles [35] and hydrogel encapsulation [36], work reasonably well in worm immobilization, they still require time-consuming manual operations. In contrast, microfluidic technology provides new possibilities for immobilization by restricting the movement of *C. elegans* and inhibiting its physiological activities [75,76,77,78,79,80,81,82,83,84,85,86,87,88,89,90]. The automated nature of microfluidic platforms eliminates the need for manual processing, saving time and resources [73,74]. Moreover, studies have shown that immobilization using microfluidic systems is mostly reversible, allowing for quick recovery of *C. elegans* [85]. Therefore, microfluidics is an ideal tool for immobilization, as it minimally affects the physiology of the worms.

Physical capture is a straightforward and non-invasive immobilization method that has been extensively explored for *C. elegans* research. One notable advantage of physical capture is its high efficiency and reversibility compared to traditional immobilization techniques. For instance, Hulme et al. introduced wedge-shaped microchannels or clamps to restrict the movement of *C. elegans*, achieving immobilization of hundreds of worms in just 15 min [75]. Worms immobilized using this approach can recover and reproduce normally, making it suitable for specific screening purposes. Similarly, Berger et al. developed a mechanical immobilization method using microchannels that match the worm’s body, ensuring the worms have access to suspended bacteria for nutrition during immobilization [76]. This approach reduces stress and minimizes potential mechanical damage. Low-pressure suction components have also been used to immobilize *C. elegans* [77]. Compressed PDMS has excellent electrostatic adsorption and bonding properties, and many researchers have utilized compressed PDMS to immobilize *C. elegans* [78,79,80]. Zeng et al. applied sudden pressure to push a PDMS membrane down, effectively wrapping *C. elegans* and achieving high immobilization efficiency and stability [79]. Gilleland et al. and Shivers et al. optimized the thickness, flexibility, and roundness of the PDMS membrane to immobilize worms while avoiding damage [81,82]. A combination of low-pressure suction components and compressed PDMS membrane, as proposed by Keil et al., allows for the immobilization of worms by first pushing them against one side of the channel due to pressure differences and then immobilizing them with the compressed PDMS membrane [83]. Gel-based immobilization techniques have also been widely used in microfluidics, utilizing electrical, optical, or other means to achieve a thermosensitive sol–gel transition that can immobilize and release worms [84,85,86]. For instance, Krajniak and Lu employed the biocompatible polymer Pluronic F127 (PF127) for worm immobilization [84]. PF127 undergoes a reversible thermosensitive sol–gel transition between 15 °C and 21 °C, making it suitable for microfluidic systems. This method is not only reversible but also does not affect the growth and development of worms at any stage due to the presence of necessary nutrients in the culture medium. Overall, physical capture methods, including microchannels, suction components, PDMS, and gel-based immobilization techniques, have demonstrated high efficiency, stability, and non-invasiveness in immobilizing *C. elegans*.

Techniques for inhibiting the physiological activities of *C. elegans* include CO_2_ anesthesia and cold/heat shock [46,87,88]. However, the potential impact of these methods on the worms’ physiology remains uncertain. Chokshi et al. designed a microfluidic device that utilizes CO_2_ permeable to a PDMS membrane to effectively inhibit the physiological activities of *C. elegans*, demonstrating its efficiency for long-term worm immobilization [87]. Additionally, Huang et al. proposed a novel immobilization method using a thick electrode to generate a uniform electric field perpendicular to the microchannel [89]. This technique allows for the efficient immobilization of *C. elegans* at any location within the microchannel, and the immobilized worms can quickly recover and resume their normal behaviors. The advantages of this approach include its non-invasiveness and the ability to immobilize multiple worms simultaneously with high precision. However, further research is needed to assess the potential impact of this method on the worms’ physiology. Similarly, Sridhar et al. discovered that surface acoustic waves (SAW) could be used to immobilize worms [90]. This non-contact method enables quick immobilization by exerting SAW acoustic force on the worms through interdigitated electrodes on piezoelectric materials. However, this device is not suitable for long-term immobilization, as it is challenging for worms to recover from the effects of the acoustic waves. Harm-free assays should be conducted to ensure the device’s harmlessness to the worms.

### 3.4. Worm-Counting Microfluidic Devices

Currently, most laboratories still rely on traditional methods for worm counting, utilizing statistical approaches and auxiliary tools, such as 64-well plates for egg counting [91]. However, as the demand for cancer detection increases, achieving high throughput in environments with large numbers of nematodes remains challenging. To address this issue, the application of artificial intelligence in image processing has made worm counting easier, solving the problem of nematode overlap [40]. Additionally, the microfluidic-based counting techniques can alleviate these worm overlap concerns [92]. Moreover, microfluidic chips are compatible with conventional micromachined sensor technology, allowing microfluidic-based devices to integrate with appropriate outreach devices, such as image recognition or capacitance sensing, thereby providing a more comprehensive range of functions [89], instead of solely sorting [71].

Microfluidic devices for nematode counting are still in the exploratory stage. In a notable study published in 2014, Zhang et al. combined microfluidics with electric impedance sensing for worm counting [92]. In this approach, as *C. elegans* passes through the measurement zone, it generates a capacitance change signal. This signal is then amplified and converted into voltage signals via specific circuits to count the worms. The electric impedance sensing technique enabled accurate and non-invasive detection of *C. elegans* that passed through microfluidic T-shape worm loading units, achieving an impressive accuracy rate of 96.1%.

## 4. Conclusions and Outlook

Regular cancer screening plays a crucial role in increasing early detection rates and reducing cancer mortality [93]. However, the high cost and invasiveness of multi-organ screening tests often deter individuals from undergoing regular screening before symptoms arise [94]. As cancer detection technologies continue to diversify, innovative approaches, such as worm-based screening, have emerged to alleviate the physical burden on people [9,14,20,21,23,95,96]. The theoretical foundation of worm-based diagnosis lies in the mechanism of nematode chemotaxis toward volatile compounds through their olfactory system. This behavioral trait is believed to be influenced by a combination of metabolites and exhibits a notable correlation with the concentration of volatile substances. Studies have indicated that certain volatiles, such as benzaldehyde and diacetyl, exert a significant chemotactic effect on nematodes within a concentration range typically spanning from 10^−2^ to 10^−3^ [19,29,97]. In breast cancer diagnosis, nematode chemotactic analysis achieves the highest accuracy with urine samples diluted to 1 in 100 [23], but the specific chemicals responsible for attracting nematodes remain unclear and require further studies. The concentrations of crucial substances in urine volatiles may approach or even dip below the olfactory sensitivity limit of the nematode, thus impeding the sensitivity of worm-based diagnosis. Besides, chemical volatility influences the behavior of nematodes, as certain compounds require sufficient time to accumulate to concentrations capable of eliciting a response from the worms [98]. A short testing time may lead to an insufficient concentration of chemotactic substances within the assay environment, whereas a long duration may result in uniform diffusion throughout the entire assay plate, thereby reducing the sensitivity and specificity of the diagnosis. Additionally, worm-based screening necessitates skilled operation and entails a time-consuming analysis process, thereby incurring high screening costs and posing challenges in validating sufficient samples. Moreover, conventional worm-based diagnosis is susceptible to external factors, such as the impact of laboratory trace odors on molecular analysis and manual operation errors, leading to high rates of false positives and false negatives. These limitations constrain the sensitivity and the specificity of the diagnosis.

*C. elegans* demonstrates the ability to detect odorant molecules emitted from cancerous urine while avoiding those present in healthy urine. However, the specific molecules responsible for this behavior remain unidentified. Previous studies have shown varying levels of certain molecules in cancerous urine compared to healthy controls [99,100,101,102,103]. For instance, in ovarian cancer, metabolites such as serine, glutamate, tyrosine, arachidonic acid, succinic acid, and acrylic acid are upregulated, while 2-phosphoenolpyruvate, benzoic acid, phenylacetic acid, nervonic acid, stearic acid, and creatinine are downregulated [104,105]. These differential metabolites may potentially activate olfactory neurons. Moreover, the neural mechanisms underlying chemotaxis in *C. elegans* remain elusive. Among the chemosensory neurons of *C. elegans*, AWA and AWC pairs guide attraction toward volatile gases, while the AWB pair elicits repulsion [106]. ASH neurons serve as polymodal nociceptors and contribute to rapid avoidance responses [106]. The subsequent validation of neural mechanisms can be conducted through biological techniques, such as calcium imaging [107,108]. Lanza et al. employed calcium imaging to demonstrate the activation of AWC neurons by cancer samples and identified a subset of G-protein-coupled receptors (GPCR) that may be involved in this neuronal response [23].

Microfluidic components present a promising solution to address these challenges by offering enhanced throughput, improved screening efficiency, and reduced staffing and material costs [69,109]. Additionally, the use of microfluidic systems minimizes physiological damage and eliminates non-experiment-related variables that often arise when working with small animal models [79,84]. Microfluidic components can be integrated into a multifunctional system for cancer screening, providing the following capabilities:

1. Microfluidic module for sorting that captures or manipulates large numbers of *C. elegans* in the L4 stage.
2. Microchannels for behavior assays that enable precise generation of concentration gradients in small areas.
3. Immobilized components that achieve high-throughput immobilization without adversely affecting the survival and reproduction of *C. elegans*.
4. Smart sensing function that alleviates the burden of manual counting and significantly enhances productivity.

In summary, microfluidic-based cancer screening using *C. elegans* shows great promise in increasing early detection rates while reducing the cost and burden on users.

However, the widespread adoption of microfluidic systems in biological laboratories has been limited by several challenges. For instance, the manufacturing process of microfluidic devices can be laborious, often requiring multiple attempts using techniques such as soft lithography to achieve successful fabrication. Furthermore, certain operations must be conducted in sterile conditions, limiting the system’s applicability. Additionally, many microfluidic devices are prone to blockages or contamination after worm-based detection, rendering them unusable and increasing overall usage costs. Nevertheless, with continuous advancements in material and manufacturing technology, these drawbacks of microfluidics are expected to be mitigated, expanding their reach. For instance, 3D printing technology holds the potential to automate the production of microfluidic components, eliminating the need for specialized knowledge in microfluidic manufacturing and overcoming existing technical challenges.

In the future, we anticipate that cancer screening processes will become more streamlined and user-friendly. Users will only be required to collect urine samples at designated medical institutions or utilize specially designed cooling bags for sample mailing. Upon receipt of the samples, the detection institution can employ integrated chips to obtain automatic results. Moreover, the design of screening devices can prioritize user-friendliness by optimizing operations that typically necessitate precise instrument control, thereby enabling manual execution. This approach would enable testing to transcend the confines of the laboratory, facilitating convenient screening in clinical settings, including hospital clinics. It also paves the way for the commercialization of screening devices, ensuring their wide-scale adoption and accessibility. As research progresses and more specific odor molecules associated with the cancer microenvironment are identified, follow-up procedures will become even more straightforward, potentially obviating the need for *C. elegans*. Instead, microfluidic integrated nanochips can directly capture volatile molecules, and mass spectrometry can indicate cancer risk. Regular follow-up visits can also be conducted, where the individual’s current physical condition and previous detection results can be inputted into machine learning programs to objectively refine the detection outcomes. Additionally, mobile phone software can be developed for health management among high-risk groups, including personalized recommendations for appropriate diets and lifestyles.

## Figures and Tables

**Table 1:** The effectiveness of the worm-based diagnosis for types of cancers.

Author (Year), Reference	Cancer Types (Patient Number)	Sensitivity (%)	Specificity (%)	Accuracy (%)	AUC ^1^	Time (min)
Kobayashi et al., 2021 [20]	pancreatic cancer (104)	84.62	60.00	72.86	NR ^2^	30–60
Kusumoto et al., 2020 [21]	pancreatic cancer (24)	NR	NR	NR	0.862	30
Kusumoto et al., 2020 [21]	colorectal cancer (67)	NR	NR	NR	0.837	30
Nishikawa et al., 2021 [22]	colorectal cancer (25)	80.00	NR	NR	NR	30–60
Kusumoto et al., 2020 [21]	gastric cancer (58)	NR	NR	NR	0.874	30
Nishikawa et al., 2021 [22]	gastric cancer (16)	68.80	NR	NR	NR	30–60
Kusumoto et al., 2020 [21]	esophageal cancer (18)	NR	NR	NR	0.870	30
Nishikawa et al., 2021 [22]	esophageal cancer (5)	80.00	NR	NR	NR	30–60
Kusumoto et al., 2020 [21]	biliary gallbladder cancer (13)	NR	NR	NR	0.893	30
Nishikawa et al., 2021 [22]	biliary tract cancer (7)	100	NR	NR	NR	30–60
Nishikawa et al., 2021 [22]	hepatocellular carcinoma (11)	90.9	NR	NR	NR	30–60
Lanza et al., 2021 [23]	breast cancer (36)	75.00	97.22	86.11	NR	60
Thompson et al., 2021 [9]	prostate cancer (21)	76.00	67.00	70.00	NR	60

^1^ AUC: area under the curve. ^2^ NR: not reported.

## Supplementary Materials

The following supporting information can be downloaded at: https://www.mdpi.com/article/10.3390/mi15040484/s1, Table S1: Summary of a standard worm-based diagnosis procedure. Table S2: Summary of microfluidic systems for high-throughput cancer screening.
