# Supplementary material for: Worm-Based Diagnosis Combining Microfluidics toward Early Cancer Screening
Source: Micromachines (Basel). 2024 Mar 31;15(4):484. doi: 10.3390/mi15040484 (PMC11052135; doi:10.3390/mi15040484)
Supplement: Supplementary file 1 [file micromachines-15-00484-s001.zip › micromachines-2904812-supplementary.pdf]

Worm-based diagnosis combining microfluidics toward early cancer screening

Yutao Shi [1], Chen Cui [1,†] ,Shengzhi Chen[1,†], Siyu Chen[1,†], Yiheng Wang[1,†], Qingyang Xu[1], Lan Yang[1], Jiayi Ye[2],
Zhi Hong[1,*] and Huan Hu [2,*]

Supplementary Materials

Table S1 . Summary of standard worm-based diagnosis procedure.

Author (year), Reference	Purpose	Advantage(s)	Disadvantage(s)
Bargmann et al, 1993[19]	Behavior Assays	Low sample needs, simple processing, and a comparatively brief testing duration.	Inaccuracies Factors like unclear boundary lines and a considerable number of immobile C. elegans
Troemel et al, 1997[28]	Behavior Assays	Reduces odor-related confusion by increasing the separation between repellent and control areas	The effects of other neurons (AWA and AWB, etc.) are not considered
Olivia et al, 2013[18]	Behavior Assays	Eliminates nematode movement bias by equidistant processing nematodes from samples and controls	Crowding may occur during movement, and still has some Inadequate design problems
Suzuki et al, 2022[29]	Behavior Assays	Anesthetics free assay allows a pure attractant or repellent response to specific.	Different altitudes of C. elegans caused by suspension or drilling to agar may trouble
Manjarrez et al., 2020[30]	Immobilization	Chemical anaesthetics can immobilize nematodes conveniently and efficiently	Nematodes require long recovery time, may damage or kill worms
Wyeth et al., 2009[32]	Immobilization	Inhibit neural activity and significantly reduced muscle contraction and behavioural activity	Might causes sustained stress responses
Robinson et al., 2016[34]	Immobilization	Short incubation of nematode at 4℃ causes severe cold shock	The cold environment damaged the tissue of nematodes and possibly fatal.
Kim et al., 2013[35]	Immobilization	Restrict nematodes while reduces direct damage to nematodes	Nematodes are unable to feed during immobilized periods
Kyra et al., 2018[36]	Immobilization	Effectively immobilized nematodes at a range of temperatures	Ultraviolet light exposure may causees minimal spectral interference and damages nematodes
Crombie et al., 2022[39]	Detection and Counting	Automatic nematode detection, counting and chemotaxis index calculation	Sediments and residues are easily misidentified thus introducing errors
Mori et al., 2022[40]	Detection and Counting	Deep neural networks can accurately identify the number of overlapping nematodes	The recall rate of one-class classification is low with only 0.79

Table S2 . Summary of microfluidic systems for high throughput cancer screening.

Author (year), Reference	Purpose	Advantage(s)	Disadvantage(s)
Dong et al., 2016[50]	Stage Sorting	L1 to L4 larvae and adult sorting and high speed of 3.5 worms per second	Impurities in the buffer may clog size-based sorting channels
Yang et al., 2017[51]	Stage Sorting	Prevents remixing after separation, significantly improving accuracy at low flow rates	The slower flow rate limits its sorting efficiency
Wang et al., 2018[52]	Stage Sorting	High sorting purity to 96% and efficiency to 2 worms per second	Electric field may have potential effects on nematodes
Atakan et al., 2020[53]	Stage Sorting	The sorting device is portable and reusable	/
Solvas et al., 2011[54]	Stage Sorting	Ultra-high sorting efficiency of 20 worms per second	Can only separate adults and larvae, cannot separate larval stages

Ai et al., 2014[55]	Stage Sorting	Not affected by nematodes' motor ability differences	Serialized device accumulates upstream errors to downstream resulting in low purity
Albrecht et al., 2011[67]	Behavior Assays	Allows for the implementation of three unique stimulation modes on C. elegans	Not entirely feasible for widespread biological assays
Yang et al., 2013[68]	Behavior Assays	enables the detection of six different compound samples quickly without anesthesia	91% accuracy is not high enough
Hu et al., 2015[69]	Behavior Assays	Reduces the influence of liquid rheological properties on C. elegans with a steady flow rate of liquid	Although concentration can be regulated, only a single chemotactic can be used
Shiga et al., 2022[72]	Behavior Assays	Enhanced the movement speed of C. elegans and successfully inhibited backward motion	Only two types of nematode are evaluated and lack of universality
Zhang et al., 2021[16]	Behavior Assays	The application of microfluidic systems for the efficient identification of metastatic cancer traits	Only could be used as a suggested reference test,
Hulme et al., 2007[75]	Immobilization	Enables the immobilization of hundreds of worms in only 15 minutes, and reversible	There are some technical flaws, such as a small number of worms stuck in the clamp
Berger et al., 2018[76]	Immobilization	Ensures that the worms have access to the nutrients, while reducing mechanical damage	Requires fine-tuning parameters
Rohde et al., 2007[77]	Immobilization	Low-pressure suction components are proposed	low stability of large scale screening due to the characteristics of nematode
Zeng et al., 2008[79]	Immobilization	Using a sudden pressure to compress a PDMS membrane encases C. elegans	There are still a few tiny movements due to not a deep anesthetic
Gilleland et al, 2010[81]	Immobilization	Adjusted the thickness, flexibility, and curvature of the PDMS membrane to reduce mechanical damage	During the process, it is easy to cause operational faults and errors
Shivers et al, 2017[82]	Immobilization	Adjusted the thickness, flexibility, and curvature of the PDMS membrane to reduce mechanical damage	There are very limited improvements to the plan Keil proposed a year ago
Keil et al, 2016[83]	Immobilization	Propose a combination of low-pressure suction components and compressed PDMS membrane	Unsuitable for research involving post-egg-laying stages or in utero embryonic development analysis.
Krajniak et al, 2010[84]	Immobilization	reversible and does not impact the growth or development of the worms at any stage	Nematodes make small shifts in position over time
Chokshi et al, 2009[87]	Immobilization	Employs a PDMS membrane permeable to CO2	The mechanism of action and potential effects of carbon dioxide are not understood
Huang et al, 2018[89]	Immobilization	Offers a non-invasive method that can immobilize multiple worms at once with great accuracy.	Further investigation is required to determine the possible effects of this technique
Sridhar et al, 2022[90]	Immobilization	Apply Surface Acoustic Wave acoustic force to the worms using interdigitated electrodes	Not ideal for extended immobilization due to acoustic wave exposure
Zhang et al, 2014[92]	Counting	Integrated microfluidics with electrical impedance sensing	Semi-automated and serial structures require some additional operational requirements
